# To see or not to see; the ability of the magno‐ and parvocellular response to manifest itself in the VEP determines its appearance to a pattern reversing and pattern onset stimulus

**DOI:** 10.1002/brb3.552

**Published:** 2016-08-25

**Authors:** Valentine L. Marcar, Lutz Jäncke

**Affiliations:** ^1^ Department of PsychologyUniversity of ZürichZürich‐OerlikonSwitzerland

**Keywords:** feedback projections, luminance contrast function, parallel processing streams, phasic and tonic neural response

## Abstract

**Introduction:**

The relationship between stimulus property, brain activity, and the VEP is still a matter of uncertainty.

**Method:**

We recorded the VEP of 43 volunteers when viewing a series of dartboard images presented as both a pattern reversing and pattern onset/offset stimulus. Across the dartboard images, the total stimulus area undergoing a luminance contrast change was varied in a graded manner.

**Results:**

We confirmed the presence of two independent neural processing stages. The amplitude of VEP components across our pattern reversing stimuli signaled a phasic neural response based on a temporal luminance contrast selective mechanism. The amplitude of VEP components across the pattern onset stimuli signaled both a phasic and a tonic neural response based on a temporal‐ and spatial luminance contrast selective mechanism respectively. Oscillation frequencies in the VEP suggested modulation of the phasic neural response by feedback from areas of the dorsal stream, while feedback from areas of the ventral stream modulated the tonic neural response. Each processing stage generated a sink and source phase in the VEP. Source localization indicated that during the sink phase electric current density was highest in V1, while during the source phase electric current density was highest in extra‐striate cortex. Our model successfully predicted the appearance of the VEP to our images whether presented as a pattern reversing or a pattern onset/offset stimulus.

**Conclusions:**

Focussing on the effects of a phasic and tonic response rather than contrast response function on the VEP, enabled us to develop a theory linking stimulus property, neural activity and the VEP.

## 1 Introduction

What I cannot create, I do not understand. Richard Feynman (1918–1988)

During the past three decades, magnetic resonance imaging has been used to map the anatomy of the neural macro‐networks serving perception and cognition and to trace their interconnections. Investigating the interactions conveyed by these interconnections requires the ability to measure change in neural activity at the millisecond level, something that is only possible using electroencephalography (EEG) or magnetoencephalography (MEG). Its low cost of acquisition and ease of use has seen EEG become the method of choice to study interactions involving neural macro‐networks. Such interactions can only be gaged from their effect on neural activity. This requires an understanding of the relationship between stimulus property, neural activity and the electric potential measured at the scalp. This however, is still a matter of uncertainty. Because the primate visual system is well understood, it serves as a model for investigating this relationship. Neural activity associated with retinal information processing is captured in the visual evoked potential (VEP), obtained by averaging the electric potential time‐locked to repeated occurrences of the event under study (Fender, Beeler, & Lehmann, 1966; Monnier & Von Berger, 1953). Its deflections are referred to as components, with each component reflecting the neural activity of a neural component; that itself may contain one or more, potentially overlapping neural processes (Luck, 2005).A linear relationship between stimulus property, neural activity and the VEP is favored by reports that the deflection amplitude of the VEP varies linearly with the diameter of a visual stimulus and hence the size of the neural population involved (Armington, 1968; Busch, Debener, Kranczioch, Engel, & Herrmann, 2004). A nonlinear relationship is favored by the observation that the sum of the VEP obtained to parts of a stimulus presented separately does not correspond to the VEP obtained to the whole stimulus (Fortune & Hood, 2003). Similarly, the VEP obtained when viewing a chequerboard as a pattern reversing stimulus differs markedly from the VEP obtained when viewing the identical chequerboard as a pattern onset stimulus. As perplexing as this observation may be, it can provide insights into the relationship between stimulus property, neural activity and the VEP.The same image pattern should result in the identical neural activity and hence give rise to VEP that are identical in appearance (Bach & Meigen, 1997; Kriss & Halliday, 1980; Shawkat & Kriss, 2000; Skandries, Richter, & Lehmann, 1980; Spekreijse, Dagnelie, Maier, & Regan, 1985). The difference in appearance of the VEP to a pattern reversing and pattern onset stimulus has been attributed to differences in the contribution of striate and extra‐striate cortex (Bach & Meigen, 1997; Dagnelie, de Vries, Maier, & Spekreijse, 1986; Di Russo et al., 2005; Jeffreys, 1970, 1971; Kelly, Schroeder, & Lalor, 2013; Kriss & Halliday, 1980; Kubova et al., 2006; Liu, Kong, Liu, & Yu, 2005; Ossenblok & Spekreijse, 1991; Spekreijse et al., 1985; Tzelepi, Ioannides, & Poghosyan, 2001; Vanni et al., 2004) or the contribution from different functional systems (Baseler & Sutter, 1997; Crewther, Brown, & Hugrass, 2016; Foxe et al., 2008; Klistorner, Crewther, & Crewther, 1997; Kromer, Serbecic, Krastel, & Beutelspacher, 2014; Pallas, Schmidt, & Dodt, 1992; Tobimatsu, Tomoda, & Kato, 1995; Zemon & Gordon, 2006; Zemon, Gordon, & Welch, 1988). Identifying the contribution of striate and extra‐striate cortex from changes in the VEP when stimulating different locations of the visual field has proven inadequate to identify the neural source (Ales, Yates, & Norcia, 2010, 2013; Kelly, Vanegas, Schroeder, & Lalor, 2013; Kelly, Schroeder, et al., 2013). Relying on the difference in contrast response function to differentiating between the contribution of the magno‐ and parvocellular system has been strongly criticized (Skottun, 2014), because the high level of anatomical interconnections within striate cortex has been argued to render this functional distinction meaningless beyond striate cortex (Kiper, Levitt, & Gegenfurtner, 1999; Nealey & Maunsell, 1994).

The ‘two‐component’ model (Victor & Zemon, 1985), developed from EEG data, builds the heart of our investigation. It divides visual processing into two independent neural components; an initial luminance component based on a mechanism selective to local luminance, followed by a contrast component based on a mechanism selective to local contrast (Victor & Zemon, 1985). Psychophysical measurements identified a phasic and a tonic mechanism contributing to visual processing (Kulikowski & Tolhurst, 1973). A phasic response and selectivity to temporal luminance contrast ($\frac{dL}{dt}$) is a characteristic of magnocellular neurons, while a tonic response and selectivity to spatial luminance contrast ($\frac{dL}{ds}$) is a characteristic of parvocellular neurons (Derrington & Lennie, 1984; Tolhurst, 1975). The involvement of different neural mechanism to the appearance of the VEP is underlined by an increase in the complexity of its structure when viewing a patterned stimulus compared to a diffuse light (Spehlmann, 1965).

We investigated the link between the neural luminance – and contrast component to the magno‐ and parvocellular system, by focussing on the phasic and tonic response characteristic of the two systems. Adult volunteers viewed a series of dartboard images, as pattern reversing and pattern onset/offset stimuli. The dartboard images were generated such that the total stimulus area undergoing a luminance contrast change was varies in a graded manner. This altered the size of the neural population activated during the neural luminance component. The identical supra‐threshold luminance contrast method was used to define the dartboard elements, we kept the discharge activity of local detectors at the same level between dartboard images. Appraising the contrast information in a dartboard image enabled us to compare the amplitude of VEP components to the size of the neural population coding its pattern. This revealed parallels between properties of the VEP and characteristics of the magno‐ and parvocellular system.

## 2 Materials and Method

### 2.1 Participants

Forty three healthy volunteers participated (23 females; mean age 26.6 years (range 18–61 years: SD: 10.2 years). None had a history of neurological illness and all had normal or corrected to normal visual acuity. All provided their written, informed consent prior to participating in the study. The protocol of the study was approved by the local ethics committee (E‐08/2006, SPUK‐ Psychiatry, Zürich, Switzerland).

### 2.2 Stimulus material

All images had a width of 1280 and a height of 1024 pixels. We generated a white disc and a series of five dartboard images with a diameter of 1020 pixels. The dartboard pattern consisted of 18 rings, each divided into 18 alternating black and white elements. We will refer to these patterns as: Disc, DB75, DB50, DB37.5, DB25 and DB12.5. We varied the ratio of white to black elements in the five dartboards so that the white elements represented 75%, 50%, 37.5%, 25% and 12.5% of the total area of the disc. We generated a complementary image of the DB50, DB37.5, DB25 and DB12.5 by rotating the original pattern by π radians. Element overlap precluded a complementary image of the DB75 being generated. Images had a 1 bit color resolution and were stored in the Microsoft bitmap format. The luminance of the white pixels was 135 cd/m^2^ that of the black pixels was .51 cd/m^2^ (Minolta: LS 110; Osaka, Japan). This corresponded to a Michelson contrast of 99.1%. At the viewing distance of 1 m, images extended from the center of gaze to an eccentricity of 8.5º. To compensate for the increase in the receptive field size with eccentricity, the size of the dartboard elements was increased with eccentricity (Rover & Bach, 1985; Zemon & Ratliff, 1982).

### 2.3 Pattern reversing stimuli

**Figure 1:**
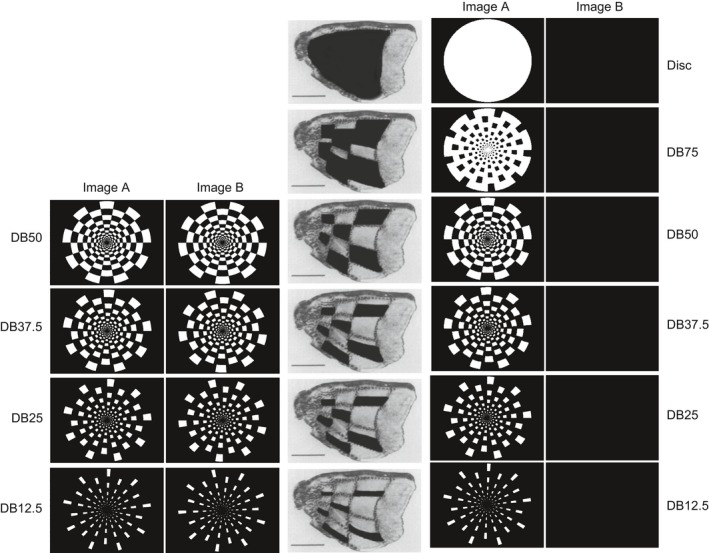
The two columns of dartboard images in the left part of the figure show the image pairs used to generate the pattern reversing stimuli. The two columns of images in the right part of the figure the images used to generate the pattern onset stimuli. The images in the middle depict the projection of the dartboard pattern onto V1. Note that overlap of dartboard elements precluded the generation of a DB75 pattern reversing stimulus

Our pattern reversing stimulus was generated using four complementary dartboard image pairs. The dartboard images used had the identical diameter but differed from each other in the stimulus area occupied by the light and dark elements. The dartboard image where 50% of the total area was occupied by the light and 50% by the dark elements was termed DB50. The dartboard image in which the total stimulus area occupied by the light elements was 37.5%, 25% and 12.5% were termed DB37.5, DB25 and DB12.5 respectively. For each dartboard image we generated a complementary version by rotating the original image by 180^°^. The characteristics of each image pair were identical, so that there was no change in mean luminance when they were exchanged. All images were presented so that the center of the image coincided with the center of the monitor. During a pattern reversal, each image was presented for 500 ms (ISI) before being exchanged by the complementary image. This resulted in two reversals per second. Each pattern reversing stimulus was presented for 60 s, generating 120 reversal events. The left half of Fig. 1 depicts the four dartboard images of the pattern reversing stimuli.

### 2.4 Pattern onset/offset stimuli

The four dartboard images used in the pattern reversing stimulus were also used in the pattern onset/offset stimuli. We added a dartboard image and a disc with identical diameter. In the dartboard image 75% of the total area was occupied by the light elements in the disc the entire area was light. The series of dartboard images thus contained a graded difference in stimulus area conveying the change in luminance contrast following pattern on‐ and offset. The pattern onset/offset stimulus was generated in the same manner as the pattern reversing stimuli, except that each dartboard image and the disc alternated with a blank image. Each pattern onset/offset stimulus was presented for 60 s, generating 60 on‐ and offset events. The right half of Fig. 1 depicts the images making up the six pattern onset stimuli.

The order of presentation of the pattern reversing and pattern onset/offset stimuli was randomized between subjects using a ‘Latin square’ procedure.

### 2.5 Apparatus

The data were recorded during two separate studies, using the identical equipment and recorded at the identical location. Recordings were performed in the laboratories of the Psychology Institute of the University of Zurich with the participant seated in a Faraday cabin (CFW, Heiden, Switzerland). Participants were instructed to keep head motion and eye blinking to a minimum and to fixate the center of the image. The stimuli were presented on a 17 inch monitor (Philips 107T4, Amsterdam, The Netherlands) using a Quadro4 700XGL graphics card (NVIDIA Corporation, Santa Clara, CA, USA). The monitor brightness was set to 50%, the contrast to 90%. Each image exchange was timed to coincide with the vertical refresh signal of the monitor, which was set to a refresh rate of 60 Hz.

#### 2.5.1 EEG recording

The scalp electric potential was recorded, using 30 Ag/Ag electrodes positioned according to the international 10/10 system (Jaspers, 1956), using an electrode cap (EasyCap GMBH, Gilching, Germany). The electrode positions used were: Fp1/2, F3/4, F7/8, Fz, FT7/8, FC3/4, FCz, T7/8, C3/4, Cz, TP7/8, CP3/4, CPz, P3/4, P7/8, Pz, O1/2, Oz. Two additional electrodes were placed below the left and right zygomatic bone to record eye movements. To minimize muscle artifacts due to chin movements, participants placed their head on a chin and forehead rest (Richmond Products Inc., Albuquerque, NM, USA). The EEG signal was recorded and stored on a workstation using the software “Vision Recorder” (Brain Products, Munich, Germany). The presentation of each image was accompanied by the placing of a unique marker in the EEG‐data.

#### 2.5.2 Postprocessing of the EEG data

The EEG data were processed off line using the software ‘Vision Analyser’ (Brain Products, Munich, Germany). EEG data was bandpass filtered removing oscillations below 0.5 Hz and above 40 Hz and set a limit on the slope of 24 dB/oct and 48 dB/oct. Blinking and muscle artifacts were identified by performing an independent component analysis (ICA) on the EEG data (T. König, University of Bern, Switzerland) and removed. During a final, visual inspection of the data, any remaining artifacts were marked manually. The signal from each electrode was re‐referenced to the average signal from all electrodes, excluding the two ocular electrodes. The epochs for each condition were located and corrected for any shift in baseline.

Working with the evoked potential provided us with an assessment of the time‐locked activity of the neural components involved (Fender et al., 1966; Luck, 2005). We focussed on the VEP from electrode Oz as it is considered to most closely reflect the activity in the striate cortex (Papakostopoulos, Hart, Corrall, & Harney, 1996; Srebro, 1987). The VEP for a specific condition was obtained by averaging the 500 ms epochs starting from the identifying marker in the EEG‐data. Epochs of image exchanges in the pattern reversing stimuli were pooled but separate VEP following image on‐ and offset of a pattern flashed stimulus were calculated.

#### 2.5.3 Pattern reversal

We identified the N75, P100 and N135 components in our pattern reversing VEP as described in the ISCEV guidelines (Odom et al., 2010). We also noted a fourth component with a positive electric potential that peaked at 240 ms. This component has been linked to perception closure (Doniger, Foxe, Murray, Higgins, & Javitt, 2002). We termed it P240. For each component and subject, we determined the peak deflection amplitude. For the N75 it was the minimum between 50–100 ms, for the P100, the maximum between 70–120 ms, for the N125, the minimum between 100–140 ms and for the P240, the maximum between 200–350 ms. The time point of the peak each component served as its latency.

#### 2.5.4 Pattern onset

The VEP we obtained matched those reported by DiRusso and colleagues (Di Russo, Aprile, Spitoni, & Spinelli, 2008). We labeled the components, C1, P1, N1 and P2. The amplitude of the C1 was the minimum in the VEP between 50–100 ms, the P1 the maximum between 80–125 ms, the N1 the minimum between 95–140 ms and the P2 the maximum between 180–350 ms. The time point of the maximum or minimum served as the component's latency.

#### 2.5.5 Pattern offset

The same parameters and nomenclature of pattern onset was used.

#### 2.5.6 Statistical analysis

We employed a mixed‐design model ANOVA with repeated measures, as implemented in the General Linear Model of SPSS Ver. 22 (IBM, Armonk, NY, USA) to analyze the amplitude and latency of the VEP components following pattern reversing, pattern on‐ and pattern offset. The within‐subject‐factors were MODE (Reversing, Onset & Offset,), AREA (Disc = 100%, DB75 = 75%, DB50 = 50%, DB37.5 = 37.5%, DB25 = 25% & DB12.5 = 12.5%) and COMPONENT (N75/C1, P100/P1, N135/N1 & P240/P2). The number of participants measured left our study overpowered. To reduce the risk of a Type I error, we rejected the NULL hypothesis if *p* ≤ .01. We followed the practice of (Victor & Zemon, 1985) and considered a response above 1 μV to be significant.

#### 2.5.7 Spatial frequency characteristics of the dartboards

**Figure 2:**
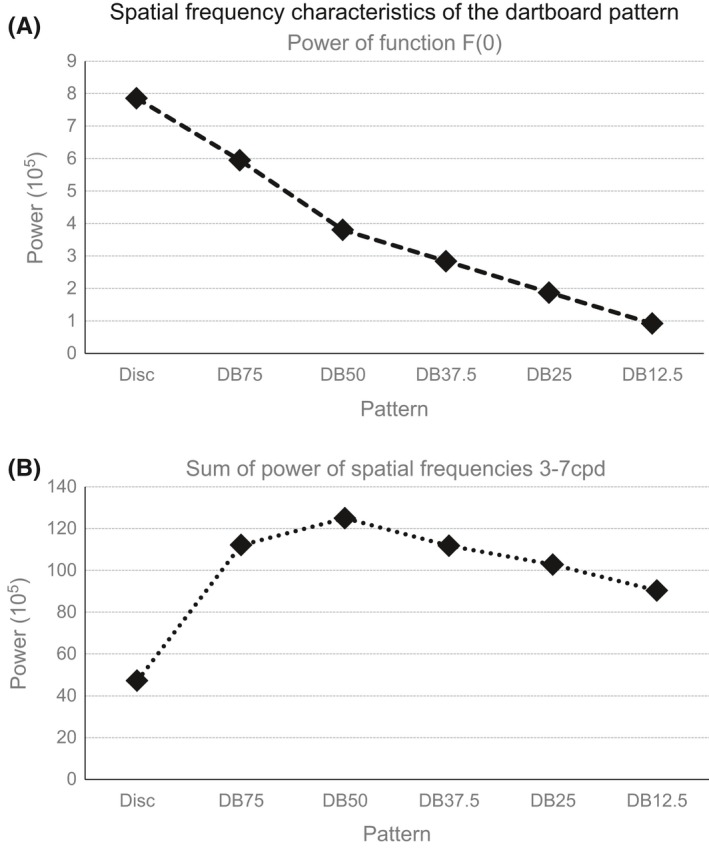
(A) The power of the function F(0) of the different stimuli; (B) the sum of the power in the spatial frequency range 3–7 cycles per degree

We determined the low and high spatial frequency characteristics of our pattern using the Fourier transformation function in MatLab, Ver. 2007a (Natick, MA, USA). The low spatial frequency characteristic was represented by the power of the function F(0) and is a measure of the proportion of light to dark elements in our images. The high spatial frequency characteristic was represented by the sum of the power in the spatial frequency range 3–7 cycles per degree (cpd), the range where human contrast sensitivity is highest (Campbell, Cooper, Robson, & Sachs, 1969; Leguire et al., 2011). It is a measure of the pervasiveness of abrupt luminance changes in the image. Figure 2 shows the low and high spatial frequency characteristic of the six patterns of our study.

#### 2.5.8 Time‐frequency power spectrum of the VEP

The time‐frequency spectrum of the VEP was obtained by performing a wavelet analysis using the ‘Wavelet’ analysis function in Analyser. The Morlet‐filter had the following parameters: Continuous Transformation; Linear interval, Absolute values and Gauss‐Borders (Standard Deviation). The resulting spectrograms are displayed in the form of a Winger plot.

#### 2.5.9 Modeling the VEP using a modified ‘two‐component’ model

**Figure 3:**
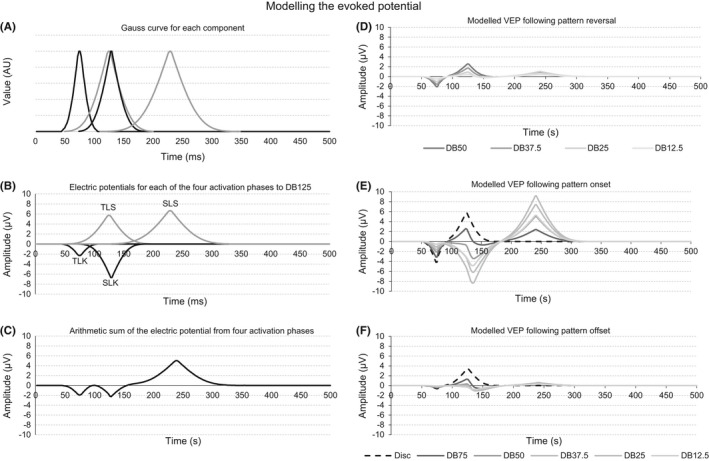
Panel A of the figure shows the Gauss functions of the four activation phases in our model. The maximum of each Gauss function is positioned to coincide with the latency of the VEP component it represents. Panel B shows the Gauss functions of Panel A, but with the Gauss function of the TLK and SLK multiplied by −1 and the TLK and TLS scaled using the power of the function F(0) of the DB25 and the SLK and SLS scaled to the sum of the power in the spatial frequencies 3–7 cycles per degree of the same dartboard image. Panel C depicts the modeled VEP obtained when the four Gauss functions of Panel B are summed. Panel D shows the modeled VEP for the four pattern reversing stimuli and Panels E and F the modeled VEP following on‐ and offset of the disc and the five dartboard images respectively

To model the VEP we adapted the ‘two‐component’ model (Victor & Zemon, 1985; Zemon & Gordon, 2006) by dividing the neural luminance and contrast component into an electric sink and source phase (See Panel A of Fig. 3). We followed Klistoner and colleagues and calculated the evoked potential associated with each activation phase (Klistorner et al., 1997) and used a Gauss function to model the electric potential of each activation phase (Rudvin & Valberg, 2006). The four activation phases were termed: ‘Temporal Luminance Sink (TLK), ‘Temporal Luminance Source’ (TLS), ‘Spatial Luminance Sink’ (SLK) and ‘Spatial Luminance Source’ (SLS). See Panel B of Fig. 3.

At a sampling rate of 500 Hz, each of our VEP contained 250 data points. The same number of data points was used in the array to model the VEP from all dartboard images during pattern reversal, pattern onset and pattern offset. The initial value of all array elements was set to zero. The number of data points in the Gauss function representing the TLK and TLS phase was double the number of data points at half maximum of the C1 and P1 in the VEP following disk onset. The number of data points in the Gauss function representing the SLK and SLS was double the number of data points at half maximum of the N1 and P2 of the VEP following the onset of the DB12.5 dartboard image. The TLK Gauss function contained 20 data points, the TLS Gauss function 40 data points, the SLK Gauss function 60 data points and the SLS Gauss function 90 data points. The peak of each Gauss function was aligned with the latency of the peak of the four VEP components. These were: 75 ms for TLK, 124 ms for the TLS, 135 ms for the SLK and 240 ms for the SLS (See Fig. 9).

The peak value of the EP associated with TLK and TLS phase of a dartboard image was obtained by first multiplying the VEP following the onset of the disc image by the ration of the power of the function F(0) of the dartboard by the power of the function F(0) of the disc, $EP_{DB}=EP_{Disc}\times \frac{F\left(0\right)\mathrm{DB}}{F\left(0\right)\mathrm{Disc}}$. The minimum in the resulting EP was taken to be the peak value of the TLK, the maximum the peak value of the TLS. To model the electric potential representing two phases was obtained by multiplying the respective peak values by the value of their respective Gauss function at each time point. The peak value of the EP associated with the SLK and SLS phase of a dartboard was obtained by first subtracting the EP from temporal luminance contrast processing of the dartboard as described above from its measured VEP. The minimum in the resulting EP was taken to be the peak value of the SLK, the maximum the peak value of the SLS. To model the electric potential of these two phases, their respective peak values were multiplied by the value of their respective Gauss function at a given time point. To model the VEP for a dartboard the four electric potentials at a given time point were added.

#### 2.5.10 Neuronal source localization

The neuronal source associated with each VEP component was identified using the electric current density (ECD) approach implemented in sLORETA‐KEY; a method that does not require any a priori assumptions to be made (Pascual‐Marqui, 2002). This method has a spatial resolution sufficient to identify the Brodman of an electric signal (MacKay, 1984). To compare the neuronal activation between stimuli the ECD was projected onto a normalized brain.

## 3 Results

### 3.1 Visual evoked potential

**Figure 4:**
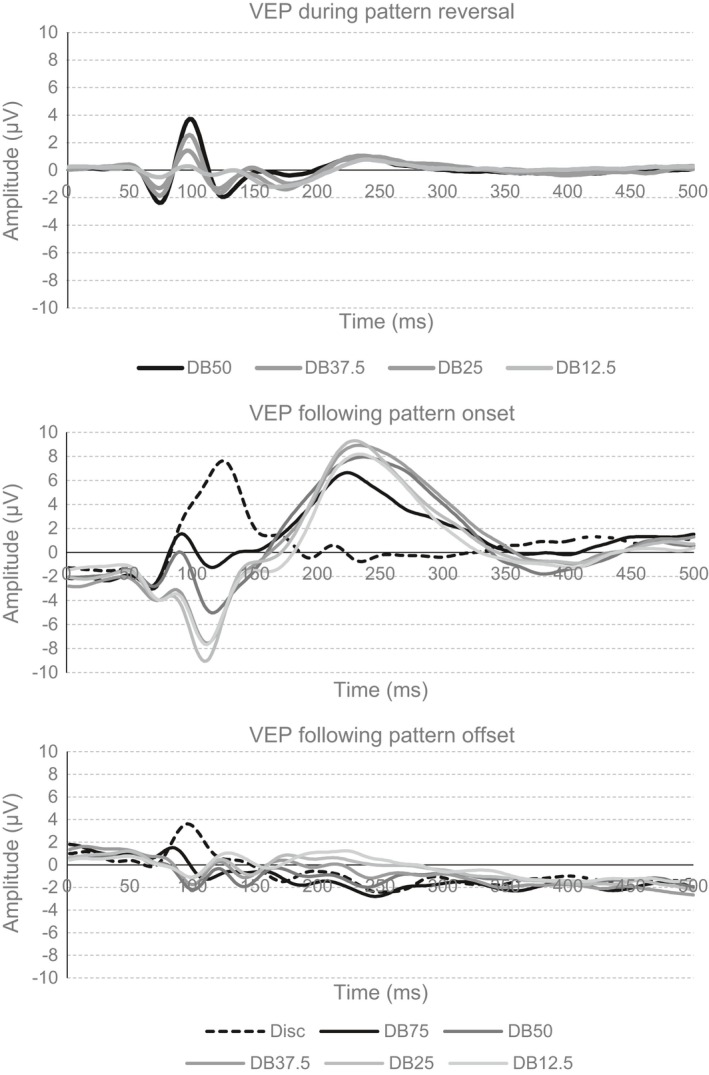
The top graph shows the VEP at Oz to the four pattern reversing stimuli. The middle graph shows the VEP at Oz following pattern onset. The bottom graph shows the VEP at Oz following pattern offset

The top panel of Fig. 4 shows the grand, mean VEP obtained to the four the pattern reversal stimuli. The leftmost of the bottom panels depicts the grand, mean VEP obtained following pattern offset in the pattern onset stimuli. The rightmost of the bottom panels shows the grand, mean VEP following pattern offset in the pattern onset stimuli.

### 3.2 Analysis of VEP component amplitude

The results of the GLM analysis of VEP component amplitude to the four dartboard images presented as pattern reversal and pattern onset/offset stimuli are given in Table 1.

**Table 1:** The table contains the result from the GLM analysis of the VEP component amplitude during the pattern reversing stimulus and following the appearance and disappearance of the disc and dartboard

Within‐Subject‐Effect	*F*	Hypothesis *df*	Error *df*	*p*	η^2^	Power
Multivariate tests (Pattern reversing, vs Pattern onset vs Offset)						
MODE	1.812	2	41	.176	.081	.155
AREA	7.834	3	40	10^−3^	.370	0.919
COMPONENT	97.295	3	40	10^−3^	.879	1.000
MODE*AREA	5.071	6	37	.001	.451	.917
MODE*COMPONENT	9.501	6	37	10^−3^	.606	.999
AREA*COMPONENT	10.646	9	34	10^−3^	.738	1.000
MODE*AREA*COMPONENT	5.811	18	25	10^−3^	.807	.998
Multivariate tests (Pattern Reversing vs Pattern Onset)						
MODE	3.658	1	42	.063	.080	.464
AREA	4.191	3	40	.011	.239	.819
COMPONENT	77.270	3	40	10^−3^	.853	1.000
MODE*AREA	9.535	3	40	10^−3^	.417	.995
MODE*COMPONENT	12.894	3	40	10^−3^	.492	1.000
AREA*COMPONENT	13.027	9	34	10^−3^	.775	1.000
MODE*AREA*COMPONENT	6.097	9	34	10^−3^	.617	.999
Multivariate Tests: N75 vs C1						
MODE	24.486	1	42	10^−3^	.368	.985
AREA	4.629	3	40	.007	.258	.655
MODE*AREA	9.901	3	40	10^−3^	.426	.974
Multivariate Tests: P100 vs P1						
MODE	6.753	1	42	.013	.139	.468
AREA	27.967	3	40	10^−3^	.677	1.000
MODE*AREA	13.037	3	40	10^−3^	.494	.996
Multivariate Tests: N135 vs N1						
MODE	14.441	1	42	10^−3^	.256	.858
AREA	8.039	3	40	10^−3^	.376	.927
MODE*AREA	8.334	3	40	10^−3^	.385	.938
Multivariate Tests: P240 vs P2						
MODE	37.373	1	42	10^−3^	.471	.999
AREA	3.495	3	40	.024	.208	.487
MODE*AREA	0.349	3	40	.790	.026	.031

Overall, the mode of presentation, i.e. pattern reversing or pattern onset, had no significant effect on the amplitude of the VEP components. There was a significant two‐way interaction between the mode of presentation and the dartboard images as well as between the dartboard images and the VEP components. There was a significant three‐way interaction between mode of presentation, dartboard image and VEP components. There was a highly significant difference in amplitude of the VEP components between dartboard images.

At the level of individual VEP components presentation mode had a significant influence on the amplitude of all but the P100/P1. Dartboard image had a significant influence on the amplitude of all but the P240/P2. A significant two‐way interaction between presentation mode and dartboard image was noted for all but the P240/P2.

#### 3.2.1 Pattern reversing

**Figure 5:**
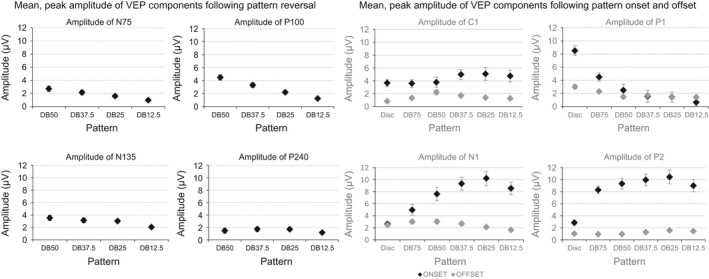
The four panels in the left half of the figure shows the mean, peak amplitude of the N75, P100, N135 and P240 obtained to the four dartboard pattern reversing stimuli. The four panels in the right half of the figure show the mean, peak amplitude of the C1, P1, N1 and P2 obtained to the disc and five dartboard images following pattern onset, dark diamonds, and following pattern offset, gray diamonds. All values shown are absolute values, to facilitate comparisons between the different VEP components. The error bars indicate the standard error of the mean (*SEM*)

Absolute values of the amplitudes and standard error of the mean of the VEP components from the four pattern reversing stimuli are shown in the four graphs in the left half of Fig. 5.

The result of the GLM analysis of the VEP component amplitude following pattern reversal are shown in Table 2.

**Table 2:** The table contains the result from the GLM analysis of the VEP component amplitude during the pattern reversing stimuli

Overall analysis						
Within‐subject‐effect	*F*	Hypothesis *df*	Error *df*	*p*	η^2^	Power
AREA	0.531	3	40	.663	.038	.045
COMPONENT	46.747	3	40	10^−3^	.778	1.000
AREA*COMPONENT	15.048	9	34	10^−3^	.799	1.000

Within‐Subject‐Contrast: COMPONENT	Trend	*F*	*df*	*p*	η^2^	Power
	Linear	10.901	1	.002	.206	.724
	Cubic	123.236	1	10^−3^	.746	1.000

Comparison of individual VEP components						
Within‐Subject‐Effect: N75	*F*	Hypothesis *df*	Error *df*	*p*	η^2^	Power
	11.722	3	40	10^−3^	.468	.91

Within‐Subject‐Contrast	Trend	*F*	*df*	*p*	η^2^	Power
	Linear	35.572	1	10^−3^	.459	.999

Within‐Subject‐Effect: P100	*F*	Hypothesis *df*	Error *df*	*p*	η^2^	Power
	28.631	3	40	10^−3^	.682	1.000

Within‐Subject‐Contrast	Trend	*F*	*df*	*p*	η^2^	Power
	Linear	85.248	1	10^−3^	.696	1.000

Within‐Subject‐Effect: N135	*F*	Hypothesis *df*	Error *df*	*p*	η^2^	Power
	8.278	3	40	10^−3^	.383	.936

Within‐Subject‐Contrast	Trend	*F*	*df*	*p*	η^2^	Power
	Linear	20.839	1	10^−3^	.332	.965

Within‐Subject‐Effect: P240	*F*	Hypothesis *df*	Error *df*	*p*	η^2^	Power
	4.514	3	40	.008	.253	.639

Within‐Subject‐Contrast	Trend	*F*	*df*	*p*	η^2^	Power
	Quadratic	12.350	1	.001	.227	.788

The amplitude of all VEP components during pattern reversal was significantly influenced by the dartboard image used. The within‐subject‐contrast indicated that the amplitude of all but the P240 VEP component varied linearly with the power of the function F(0). The amplitude of the P240 exhibited a quadratic relationship with the same measure).

#### 3.2.2 Pattern onset/offset

Absolute values of the amplitudes and standard error of the mean of the VEP components following pattern onset/offset are shown in the four graphs in the right half of Fig. 5.

The results of the GLM analysis of the VEP component amplitudes following pattern onset are listed in Table 3.

**Table 3:** The table contains the result from the GLM analysis of the VEP component amplitude following the appearance of the disc or dartboard image (Patten onset)

Overall comparison						
Within‐Subject‐Effect	*F*	Hypothesis *df*	Error *df*	*p*	η^2^	Power
AREA	5.517	5	38	.001	.421	.910
COMPONENT	61.995	3	40	10^−3^	.823	1.000
AREA*COMPONENT	11.757	15	28	10^−3^	.863	1.000

Within‐Subject‐Contrast	Trend	*F*	*df*	*p*	η^2^	Power
AREA	Linear	26.851	1	10^−3^	.390	.992
	Quadratic	10.261	1	.003	.196	.692
	Cubic	7.369	1	.010	.149	.513
COMPONENT	Linear	37.279	1	10^−3^	.470	.999
	Quadratic	9.892	1	.003	.191	.672
	Cubic	120.061	1	10^−3^	.741	1.000

Comparison of individual VEP components						
Within‐Subject‐Effect: C1	*F*	Hypothesis *df*	Error *df*	*p*	η^2^	Power
	9.447	5	38	10^−3^	.554	1.000

Within‐Subject‐Contrast	Trend	*F*	*df*	*p*	η^2^	Power
	Linear	12.843	1	10^−3^	.234	.990
	Quadratic	15.394	1	10^−3^	.268	.969

Within‐Subject‐Effect: P1	*F*	Hypothesis *df*	Error *df*	*p*	η^2^	Power
	8.604	5	38	10^−3^	.531	.999

Within‐Subject‐Contrast	Trend	*F*	*df*	*p*	η^2^	Power
	Linear	16.220	1	10^−3^	.279	.976

Within‐Subject‐Effect: N1	*F*	Hypothesis *df*	Error *df*	*p*	η^2^	Power
	7.420	5	38	10^−3^	.494	.997

Within‐Subject‐Contrast	Trend	*F*	*df*	*p*	η^2^	Power
	Quadratic	28.442	1	10^−3^	.404	.999

Within‐Subject‐Effect: P2	*F*	Hypothesis *df*	Error *df*	*p*	η^2^	Power
	5.444	5	38	.001	.417	.905

Within‐Subject‐Contrast	Trend	*F*	*df*	*p*	η^2^	Power
	Quadratic	17.825	1	10^−3^	2.98	.930
	Cubic	8.047	1	.007	.161	.559

The amplitude of all VEP components following pattern onset was significantly influenced by the dartboard image used. Analysis of individual components indicated that the amplitude of all but the P1 VEP component exhibited a linear and quadratic relationship with the power of the function F(0). The amplitude of the P1 exhibited a linear relationship only, while the P2 also exhibited a cubic relationship with this stimulus property.

The results of the analysis of VEP component amplitude following pattern offset are listed in Table 4.

**Table 4:** The table contains the result from the GLM analysis of the VEP component amplitude following the disappearance of the disc or dartboard image (Pattern offset)

Overall comparison						
Within‐Subject‐Effect	*F*	Hypothesis *df*	Error *df*	*p*	η^2^	Power
AREA	4.312	5	38	.003	.362	.795
COMPONENT	67.164	3	40	10^−3^	.834	1.000
AREA * COMPONENT	5.447	15	28	10^−3^	.745	.996

Within‐Subject‐Contrast	Trend	*F*	*df*	*p*	η^2^	Power
AREA	Quadratic	11.275	1	.002	.212	.742
	Cubic	17.963	1	10^−3^	.300	.932
COMPONENT	Linear	36.291	1	10^−3^	.464	.999
	Cubic	191.924	1	10^−3^	.820	1.000

Comparison of individual VEP components						
Within‐Subject‐Effect: C1	*F*	Hypothesis *df*	Error *df*	*p*	η^2^	Power
	3.854	5	38	.006	.336	.904

Within‐Subject‐Contrast	Trend	*F*	*df*	*p*	η^2^	Power
	Quadratic	9.004	1	.005	.177	.843

Within‐Subject‐Effect: P1	*F*	Hypothesis *df*	Error *df*	*p*	η^2^	Power
	7.770	5	38	10^−3^	.506	.998

Within‐Subject‐Contrast	Trend	*F*	*df*	*p*	η^2^	Power
	Linear	31.492	1	10^−3^	.429	1.000

Within‐Subject‐Effect: N1	*F*	Hypothesis *df*	Error *df*	*p*	η^2^	Power
	3.539	5	38	.010	.318	.875

Within‐Subject‐Contrast	Trend	*F*	*df*	*p*	η^2^	Power
	Quadratic	9.172	1	.004	.179	.841
	Cubic	10.290	1	.003	.197	.880

The amplitude of all VEP components except the P2 were significantly influenced by the dartboard image used. Analysis of individual components found a linear relationship between the power of the function F(0) of the dartboard and the P1. The C1 and N1 exhibited a quadratic relationship with this stimulus property.

### 3.3 Analysis of VEP component latency

The results of the statistical analysis of the VEP component latencies following pattern reversal, pattern onset and pattern offset are shown in Table 5.

**Table 5:** The table contains the result from the GLM analysis of the VEP component latencies during the pattern reversing stimuli

Overall comparison						
Within‐Subject‐Effect	*F*	Hypothesis *df*	Error *df*	*p*	η^2^	Power
AREA	2.779	3	40	.053	.172	.624
AREA*COMPONENT	0.807	9	34	.613	.176	.326

Overall, no difference in latency of any VEP component across the dartboard images presented as pattern reversing and pattern onset stimuli was observed. The latencies obtained following pattern onset had a significant interaction AREA*COMPONENT. A significant increase in latency of the C1 and significant decrease in latency of the N1 was observed with the power of the function F(0) of the dartboard image. Overall, the factor AREA exerted a significant influence on the latencies of the VEP components obtained following pattern offset. The interaction AREA*COMPONENT was not significant.).

### 3.4 Time‐frequency power analysis of the VEP

#### 3.4.1 Pattern reversing

**Figure 6:**
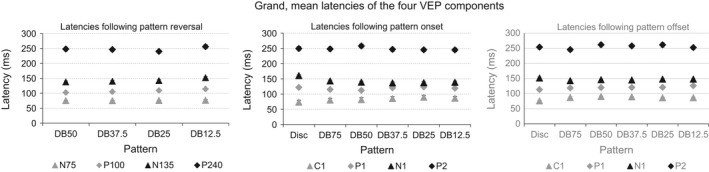
The leftmost panel shows the grand, mean latency of the N75, P100, N135 and P240 to the pattern reversing stimuli. The middle panel shows the grand, mean latency of the C1, P1, N1 and P2 following pattern onset. The rightmost panel shows the grand, mean latency of the C1, P1, N1 and P2 following pattern offset. The error bars indicate the standard error of the mean (*SEM*)

The time‐frequency power spectrogram of the VEP following pattern onset is shown as Winger plots in the two left most columns of Fig. 6.

The analysis showed that during neural processing of temporal luminance contrast frequencies in the ß‐band dominated the oscillation in the VEP, while during neural processing of spatial luminance contrast frequencies in the α‐band dominated the oscillations of the VEP (Table 6).

**Table 6:** The table contains the result from the GLM analysis of the VEP component latensies following onset and offset of the disc and dartboard images

Pattern onset						
Within‐subject‐Effect	*F*	Hypothesis *df*	Error *df*	*p*	η^2^	Power
AREA	0.705	5	38	.623	.085	.227
AREA * COMPONENT	3.829	15	28	.001	.672	.993

Comparison of individual components						
Within‐Subject‐Effect: C1	*F*	Hypothesis *df*	Error *df*	*p*	η^2^	Power
	4.641	5	38	.002	.379	.953

Within‐Subject‐Contrast	Trend	*F*	*df*	*p*	η^2^	Power
	Linear	20.767	1	10^−3^	.331	.994

Within‐Subject‐Effect: P1	*F*	Hypothesis *df*	Error *df*	*p*	η^2^	Power
	2.081	5	38	.089	.215	.625

Within‐Subject‐Effect: N1	*F*	Hypothesis *df*	Error *df*	*p*	η^2^	Power
	3.939	5	38	.006	.341	.911

Within‐Subject‐Contrast	Trend	*F*	*df*	*p*	η^2^	Power
	Linear	9.943	1	.003	.191	.869

Within‐Subject‐Effect: P2	*F*	Hypothesis *df*	Error *df*	*p*	η^2^	Power
	0.889	5	38	.498	.105	.282

Pattern offset						
Within‐Subject‐Effect	*F*	Hypothesis *df*	Error *df*	*p*	η^2^	Power
AREA	4.269	5	38	.004	.360	.934
AREA*COMPONENT	2.361	15	28	.024	.559	.906

Within‐Subject‐Contrast	Trend	*F*	*df*	*p*	η^2^	Power
AREA	Linear	13.998	1	.001	.250	.955

#### 3.4.2 Pattern onset

The Winger plots of the VEP following pattern onset are shown as in the two left most columns of Fig. 6. The Winger plots show that oscillation frequencies in the ß‐band dominate the VEP during temporal luminance contrast processing, while oscillations in the α‐band dominated the VEP during spatial luminance contrast processing. Oscillations in the δ‐band dominated the late stages of spatial luminance contrast processing of the DB75, DB50 and DB37.5.

#### 3.4.3 Pattern offset

The Winger plots of the VEP following pattern offset are shown as in the two right most columns of Fig. 6. Oscillation in the β‐band dominate VEP during temporal luminance contrast processing, while oscillations in the β‐band dominate the VEP during spatial luminance contrast processing of the dartboard but not the disc image. As during pattern onset oscillation in the δ‐band emerged during the late stages of spatial luminance contrast processing.

### 3.5 Neural source localization

#### 3.5.1 Pattern reversing

**Figure 7:**
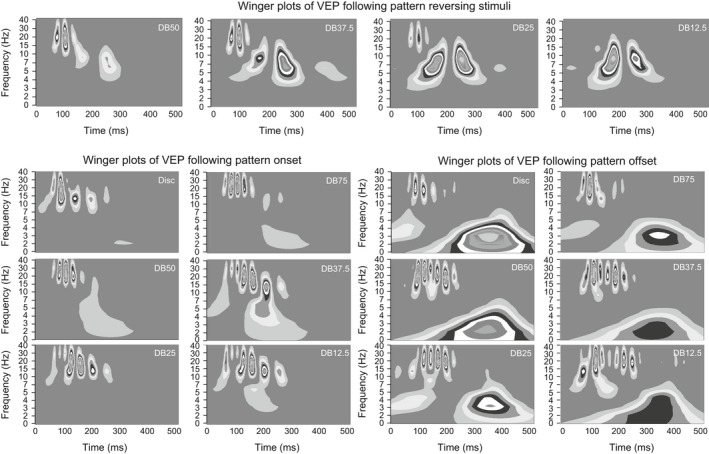
The panels in the top row of the figure depict Winger plots of the time‐frequency distribution in the VEP obtained from the four pattern reversing dartboard stimuli. The six panels in the left half of the lower part of the figure depict Winger plots of the VEP obtained following pattern onset. The six panels in the right half of the lower part of the figure depict Winger plots of the VEP obtained following pattern offset

Figure 7 shows that the electric current density (ECD) during the N75 and N135 VEP component was highest at the occipital pole; the location of striate cortex. During the P100 the highest ECD was located in posterior parietal cortex, specifically in Cuneus and Precuneus. During the P240 the overall ECD distribution resembled that observed during the N135 except that it was more diffuse.

#### 3.5.2 Pattern onset

**Figure 8:**
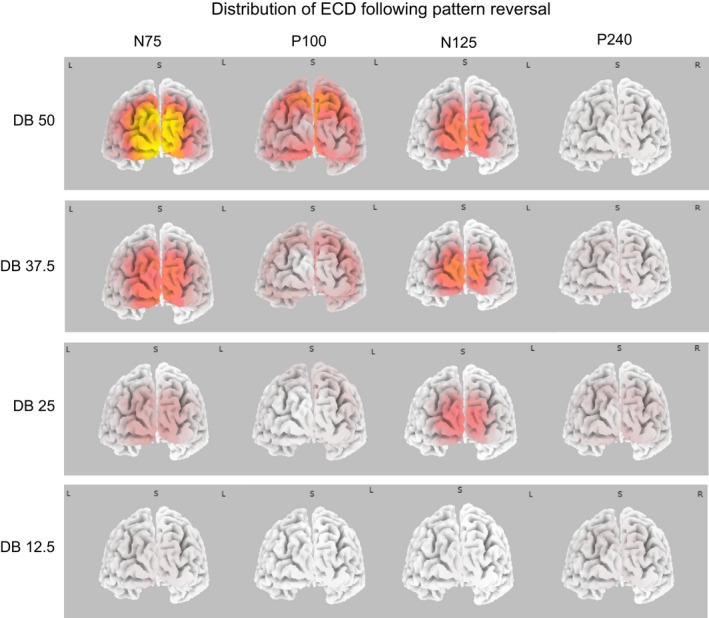
The four panels depict the distribution of the cortical electric current density (ECD) for each VEP component as obtained to the four dartboard pattern reversing stimuli. The left most brain image shows the ECD associated with the N75, the left of middle brain image the ECD associated with the P100, the right of middle brain image the ECD associated with the N135 and right most image the ECD associated with the P240. During the N75 and N135 ECD was highest at the occipital pole, while during the P100 and P240 it is highest in the surrounding area

The panels in the bottom, left half of Fig. 8 show the ECD distribution following pattern onset. The Disc lacked any spatial contrast except at its periphery. This accounts for the VEP it generated being simpler than that obtained to the dartboard stimuli (Spehlmann, 1965), as it lacked the N1 and P2 observed to a dartboard pattern.

During the C1 ECD was highest at the occipital pole for all patterns presented. During the P1 ECD was highest in posterior parietal cortex. The ECD was weaker to the Disc, DB75 and DB50 than to the DB37.5, DB25 and DB12.5. During the N1 ECD to the DB37.5 and DB25 was weaker than that to the DB75, DB50 and DB12.5 and highest at the occipital pole and posterior parietal cortex. The ECD during the P2 was lower than during the P1 and highest in extra‐striate cortex.

#### 3.5.3 Pattern offset

The six panels in the right, bottom half of Fig. 8 show the distribution of the ECD across cortex following pattern offset. The ECD following pattern offset was lower than following pattern onset. During the C1 the ECD was observable following Disc and DB37.5 offset and highest ECD located in parietal cortex. During the P1 following Disc offset, the ECD was highest in the Cuneus. During the P1 following DB75 offset the ECD was highest in posterior parietal cortex and the region surrounding the pole of the occipital lobe. In the remaining dartboard pattern the ECD was highest in posterior parietal cortex. During the N1 following offset of a dartboard the ECD was highest in the posterior parietal cortex. During the P2 the ECD was highest in the region surrounding the pole of the occipital lobe.Source localization revealed that activity in striate cortex coincided with the electric source, activity in extra‐striate cortex with the electric sink in the VEP.

### 3.6 Model of the visual evoked potential

**Figure 9:**
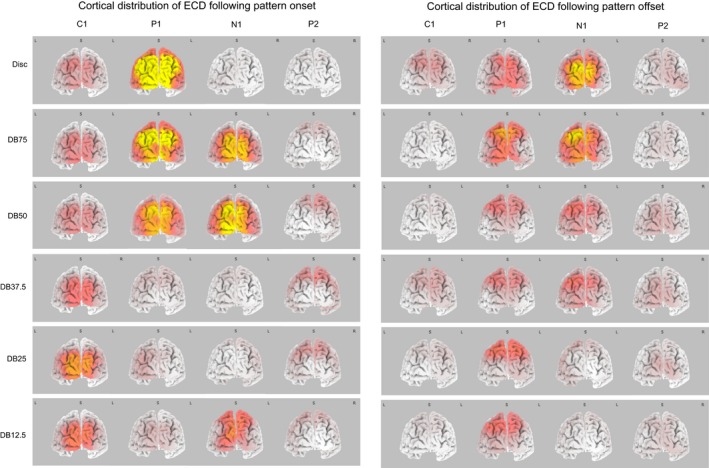
The six panels in the left half of the figure depict the cortical distribution of the ECD associated with the C1, P1, N1 and P2 following pattern onset. The panels in the right half of the figure depict the cortical distribution of the ECD for the same components following pattern offset. For the dartboard images, the highest ECD during the C1 and N1 was observed at the occipital pole, while during the P1 and P2 the ECD was highest in parietal cortex. For the disc image the highest ECD during the C1 and P1 was observed at the occipital pole

Panel A of Fig. 9 depicts the Gauss distributions used to model the electric potential of TLK, TLS SLK and SLS. Panel B shows the modeled electric potential of each activation phase to onset of the dartboard DB50 stimulus. Panel C depicts the modeled VEP derived from the electric potentials shown in Panel B. Panel E to F depict the modeled VEP to the pattern reversing, pattern onset and pattern offset respectively.

## 4 Discussion

### 4.1 Summary of findings

Differences in amplitude of VEP components between our dartboard images, when viewed as pattern reversing and pattern onset/offset stimuli pointed to the presence of a phasic and tonic neural response. The amplitude of the N75, P100 and N135 from our dartboards viewed as pattern reversing stimuli mirrored the total dartboard area undergoing a luminance contrast change, indicating neural mechanism selective to temporal luminance contrast. The amplitude of the P240 from our pattern reversing stimuli as well as those of the C1, N1 and P2 following pattern onset had a quadratic relationship with the area in the dartboard images undergoing a luminance contrast change. This indicated neural mechanism selective to spatial luminance contrast.

Significant differences in the latency of the C1 and N1 following onset and offset of the disc were observed compared to the same components following onset and offset of the dartboard pattern. The time‐frequency spectrogram of the VEP to the pattern reversing stimuli, revealed oscillations frequencies in the beta range during the initial VEP followed by oscillations frequencies in the alpha range. The time‐frequency spectrogram of the VEP following pattern onset and offset revealed oscillation frequencies in the beta range during the initial 200 ms of the VEP followed by weak oscillations with frequencies in the alpha range. During the N75/C1 and N135/N1 the electric current density was highest at the occipital pole, while during the P100/P1 and P240/P2 the electric current density was highest in the region surrounding striate cortex.

Based on our findings we will argue that the appearance of the VEP to a pattern reversing and pattern onset stimulus is a governed by the ability of EEG to register the phasic and tonic neural response. We will further discuss parallels between amplitude and temporal frequency characteristic of the VEP and the magno‐ and parvocellular system, that link the phasic neural response to the former and the tonic neural response to the latter. Before this we will dismiss diploe cancellation, the asymmetry in the neural response of the ON and OFF system and a difference in a neural motion signal as explanations for our findings.

### 4.2 The influence of dipole cancellation, ON and OFF system and motion signal on the VEP

The ‘Cruciform model’ envisages electric dipoles of opposite polarity when the upper and lower bank of calcarine sulcus are activated (Jeffreys, 1971; Vanni et al., 2004). Its usefulness to differentiate between striate and extra‐striate activity has recently been called into question (Ales et al., 2010, 2013; Kelly, Schroeder, et al., 2013; Kelly, Vanegas, et al., 2013). As our dartboard images activated both upper and lower bank of calcarine sulcus concurrently, dipole cancellation needs to be exclude as an account for our findings. Given the symmetry in the dartboard images, the electric dipoles resulting from activation of both upper and lower bank of calcarine would have canceled each other and nulled the VEP. This would have applied to all our images whether presented as a pattern reversing pattern or pattern onset stimulus. With an amplitude of 9 μV to a full‐field dartboard pattern, dipole cancellation can be excluded as an explanation. Our VEP also compares favorably to the amplitude of 7 μV obtained to the full‐field, chequerboard pattern used in the study from which the two‐component model was developed (Victor & Zemon, 1985).

Asymmetry in the response of the independent on‐ and off‐systems in the primate visual (Harris & Parker, 1995; Shawkat & Kriss, 2000; Zemon & Gordon, 2006) may account for the difference in the VEP to pattern reversing and pattern onset/offset stimuli. The former activated the two systems concurrently, while the latter activated them consecutively. The lower amplitude of the VEP components from the pattern reversing stimuli implied the presence of destructive interference. As the sequence of polarity reversals in the VEP following pattern on‐ and offset was identical, they could only have interfered constructively; leading us to dismiss an interaction between the on‐ and off‐system as an explanation.

The presence of a neural motion signal during a pattern reversing but not during a pattern onset stimulus has frequently been cited to account for the difference in the VEP (Dagnelie et al., 1986; Kobayashi, Yoshino, Kawamoto, Takahashi, & Nomura, 2004; Kubova, Kuba, Spekreijse, & Blakemore, 1995; Spekreijse et al., 1985). Based on the influence of changes in luminance contrast, the neural motion signal has been linked to the deflection in the VEP with a negative electrical polarity, occurring between 160–200 ms (Bach & Ullrich, 1997; Kubova et al., 1995). The difficulty with a difference in a neural motion signal as an explanation is that the VEP provides insight into processing mechanisms rather than information processed (Mitzdorf, Li, & Poppel, 1994). In the spatiotemporal energy model of motion perception, the first‐order motion signal is derived from the quotient of the signal from the temporal – ($\frac{dL}{dt}$) and spatial luminance contrast ($\frac{dL}{ds})$ mechanism. Speed (*v*) is given by $v=\frac{dL}{dt}/\frac{dL}{ds}=\frac{dLds}{dtdL}=\frac{ds}{dt}$ (Adelson & Bergen, 1985; Hildreth & Koch, 1987; van Santen & Sperling, 1984). The projected influence of a motion signal on the VEP, may therefore arise from a change in the interaction between the temporal – and spatial luminance contrast channels at different luminance contrast rather than a separate motion signal. While not resolving this issue, we offer an alternative involving established processing mechanisms.

We will next outline the neural mechanism of the VEP and present a brief summary of the anatomical and functional organization of the primate visual system.

### 4.3 The VEP as a measure of neural activity and an outline of the primate visual system

The VEP is a product of the ionic current flowing between the apical dendrites and the soma of pyramidal cells; a current generated by a change in the local field potential, resulting from all excitatory and inhibitory post‐synaptic action potentials acting at the apical dendrites (Creutzfeldt, Rosina, Ito, & Probst, 1969; Lehmann & Skrandies, 1982). Deflections in the VEP reflect the discharge activity of all neurons responding to a stimulus rather than the discharge activity of a specific neural population (Celesia, 1993). The linear increase in amplitude of the VEP with the size of the neural population responding to a stimulus (Armington, 1968; Busch et al., 2004) confirms the linear relationship between the VEP and neural activity (Lehmann & Skrandies, 1982).

Anatomically, the primate visual system is divided into striate and more than 30 extra‐striate visual areas (Van Essen, 1979); each making a specific contribution to the perception of form, color and motion. Though extensively interconnected they congregate into dorsal stream terminating in the parietal lobe and a ventral stream terminating in the temporal lobe. The former serves motion perception and the perception of spatial relationships. The latter serves color and form perception. Areas of the dorsal stream contain a retinotopically organized representation of at least part of the contralateral visual field (Brewer, Press, Logothetis, & Wandell, 2002), while in the ventral stream a retinotopic organization gives way to an object centered organization (Tanaka, 1993).

Functionally, the primate visual system is based on the input from three systems, the magno‐, parvo‐ and koniocellular system (Briggs & Usrey, 2011; Seki et al., 1996), with only the latter two conveying chromatic information (Kulikowski, Robson, & Murray, 2002). The koniocellular system responds too poorly to an achromatic stimulus, even at high luminance contrast (Gouras, Mackay, Roy, & Yamamoto, 1993) so that its contribution to the VEP in our study can be considered negligible. The number of parvocellular fibers carried by the optic nerve/tract exceeds the number of magnocellular fibers by a factor between eight (Dacey, 1993) and thirty‐five (Azzopardi, Jones, & Cowey, 1999). The magnocellular system is based on a temporal luminance contrast ($\frac{dL}{dt}$) mechanism (Robson, 1966; Tolhurst, 1975), responds in a phasic manner to a stimulus and its response saturates at a luminance contrast above 16–30% (Derrington & Lennie, 1984). The parvocellular system is based on a spatial luminance contrast ($\frac{dL}{ds}$) mechanism, responds in a tonic manner to a stimulus and its response increases linearly with increasing luminance contrast. Most magnocellular neurons feed into the dorsal processing stream, with a minority entering the ventral processing stream (Merigan & Maunsell, 1993), whereas all parvocellular neurons enter the ventral stream. Axons of magnocellular neurons have a faster conduction velocity than those of the parvocellular neurons. At 50 ms, the magnocellular signal arrives in striate cortex 20 ms ahead of the parvocellular signal (Foxe & Simpson, 2002), a time difference referred to as the ‘magnocellular advantage’ (Klistorner et al., 1997; Laycock, Crewther, & Crewther, 2007). Because feedback projections modulate activity in V1 of the monkey within 10 ms (Hupe et al., 2001), all VEP components in our study reflect neural activity modulated by feedback projections.

### 4.4 On the difference in the VEP to our pattern reversing and pattern onset/offset stimuli

During the 500 ms each images was presented, the phasic neural response will have subsided before the next image exchange while the tonic neural response will have persisted. The tonic neural response will thus be present as a DC shift in the scalp electric potential, something the EEG is insensitive to. Consequently, although our pattern reversing stimuli elicited both a phasic and tonic response, only the former will be manifest in the VEP. The change from a linear relationship in the amplitude of the N75, P100 and N135 and the dartboard area undergoing a luminance contrast change to a quadratic one of the P240 would be explained by the waning of the phasic response between 135 ms and 240 ms after an image exchange, leaving the P240 reflecting tonic neural response dampened by a ceiling effect.

Following pattern offset the tonic response will subside and with it the ionic current it generates. As a result the VEP following pattern onset will have comprised both the phasic and tonic neural response, which would explain the larger amplitude of the C1, N1 and P2 compared to the N75, N135 and P240.

In areas with a retinotopic organization differences in total area stimulated corresponds to differences in the number of neurons stimulated. The linear relationship between the amplitude of the N75, P100 and N135 and the dartboard area undergoing a luminance contrast change, therefore links these to the size of the neural population activated. With the phasic neural response during temporal luminance processing modulated by feedback projections, these must have left the distribution of the activated neurons across V1 unaltered. Because feedback projections can activate neurons in the absence of a forward signal (Chen et al., 2007; Givre, Schroeder, & Arezzo, 1994; Schroeder, Mehta, & Givre, 1998) an alternative to the multiplicative nature of feedback projections (Allman, Miezin, & McGuinness, 1985; Fukushima, 1988; Grossberg, 1999) is required. The narrower dispersion of feedback projections between visual areas containing a retinotopic organization compared to between areas lacking such an organization (Blasdel, Lund, & Fitzpatrick, 1985) represents an alternative. Only areas of the dorsal stream contain a retinotpoic representation of at least part of the contralateral visual field (Brewer et al., 2002), feedback projections modulating the neural response during temporal luminance processing most likely originated in areas of the dorsal stream. This possibility is supported by the presence of higher temporal frequency oscillation in the electric potential during temporal luminance processing than during spatial luminance processing. High temporal frequency oscillations have been associated the neural processing of low spatial frequency content of a stimulus (Frund, Busch, Korner, Schadow, & Herrmann, 2007). Both low (von Stein, Chiang, & Konig, 2000; von Stein & Sarnthein, 2000) and high temporal frequency oscillation in the electric potential (Makeig et al., 2002; Singer, 1993) have been associated with interactions between neural systems. Because activation latencies of visual areas along the dorsal stream are shorter than those along the ventral stream (Chen et al., 2007; Schroeder et al., 1998), interactions with areas of the dorsal processing stream will therefore be faster and the resulting oscillation frequencies in the electric potential higher than those resulting from interactions with areas of the ventral processing stream.

### 4.5 Final issues

In this section we will examine three observation of the VEP that we have found difficult to reconcile with the functional and anatomical characteristics of the primate visual system. The first is that the amplitude to the four VEP components obtained to our pattern onset stimuli were considerably larger than those obtained to our pattern reversing stimuli. The same was the case with the amplitude of the P1 following the onset of the disc and the amplitude of the P2 following onset of a dartboard image. In both cases processing the temporal luminance contrast was expected to involve a larger neural population than processing the spatial luminance contrast.

The first explanation for this is that the power in the function F(0) and the spatial frequency range 3–7 cpd corresponds to the size of the neural population required to code them (See Fig. 3). The second is synchronization of activity. Synchronizing 10% of a neural population increases the amplitude of the electric potential by an order of magnitude (Elul, 1971). The issue with both of these explanation is that the mismatch between the measured and expected difference in component amplitude. We observed a doubling in amplitude, while the magno‐ to parvocellular ratio, while the effect of synchronizing neural activity predicts a difference in the order of magnitude.The second issue that the DB50 dartboard image had the highest power in its high spatial frequency range, yet did not generate the largest amplitude in N1 and P2. The largest amplitude in these components was elicited by the DB25 dartboard image: the dartboard with the least elongated elements. This mismatch can be understood by considering the inhibitory effects of end‐stopping on the neural response. Associated with single cell activity, end‐stopping has been shown to influence psychophysical threshold (Yu & Levi, 1997) and the VEP (Polat & Norcia, 1998).

The third and last issue is the neural basis of the sink and source phase in the VEP during the two processing stages. Kubova and colleagues attributed negative deflections to processing by the magnocellular and positive deflections to processing by the parvocellular system (Kubova et al., 1995). Our findings do not corroborate this view. This explanation runs contrary to the polarity of a VEP deflection being determined by the direction of flow of the ionic current between apical dendrite and soma of pyramidal cells (Creutzfeldt et al., 1969; Lehmann & Skrandies, 1982). A reversal in the direction of flow of the ionic current is initiated by the inhibition of neural activity (Creutzfeldt et al., 1969) and blocking GABA receptors in striate cortex of the monkey with the GABA antagonist Bicuculline, abolishes the P80 (Kraut, Arezzo, & Vaughan, 1990; Zemon, Kaplan, & Ratliff, 1980). A reversal in the polarity of a deflection in the VEP has also been linked to a shift in the neural activity between the lamina of V1 (Gilbert & Wiesel, 1979). Studies involving the monkey have linked the N40, the monkey equivalent of the N75, to activity in the granular layer of V1 and the P80, the monkey equivalent of the P100, to activity in its supragranular layers. Later VEP component could not be reliably linked to laminar activity (Givre et al., 1994; Schroeder et al., 1998). The last two mechanisms need not be exclusive as the former may be the basis of the latter. These studies support our view that the sink and source phase during the phasic response based on a mechanism selective parallels a shift in neural activity toward the supragranular layers followed by a shift in activity toward the infragranular layers. Evidence that the same applies for neural activity attributed to the tonic response based on a mechanism selective to spatial luminance contrast is currently not available. Reports of a contribution of V1 to different processing stages do not exclude this possibility (Roelfsema, Tolboom, & Khayat, 2007).

## 5 Conclusions

The findings of our study led us to the conclusion that the difference in the appearance of the VEP to a pattern reversing and pattern onset stimulus does not arise from a difference in neural response. Instead it arises from the ability of the neural response driven by the magno‐ and parvocellular system to manifest itself in the VEP. It is therefore the stimulus paradigm itself that is the origin of the difference in the appearance of the VEP to a pattern reversing and pattern onset stimulus. While extensive interconnection within striate cortex renders the distinction between a neural response driven by the magno‐ and parvocellular system meaningless beyond striate cortex, other physiological and response characteristics of the two systems remain evident in the neural response during visual processing.

By considering the phasic and tonic response properties and the temporal – and spatial luminance contrast selectivity of the magno‐ and parvocellular system, we were able to link characteristics of or dartboard images and the VEP to the size of the neural population contributing to the neural response at different processing stages. The model drawing on the neural response characteristics of the magno‐ and parvocellular will enable future research to develop testable predictions of the effect stimulus property on the appearance of the VEP.

## Funding Information

None.

## Conflict of Interest

None declared.
